# Domestic

**Published:** 1841-11-06

**Authors:** 


### DOMESTIC.

### HEALTH OF THE CITY.

INTERMENTS *in the City and Liberties of Philadelphia, from the 23d of October to the 30th.*

| Diseases. | Adults. | Children. | Diseases. | Adults. | Children. |
|---|---|---|---|---|---|
| Apoplexy | 3 | 0 | *Brought forward,* | 42 | 34 |
| Burns, | 1 | 0 | Marasmus, | 0 | 4 |
| Cancer, | 1 | 0 | Measles, | 0 | 1 |
| —— Womb, | 1 | 0 | Menorrhagia, | 1 | 0 |
| Casualties, | 0 | 2 | Mania a Potu, | 1 | 0 |
| Croup, | 0 | 2 | Morbus Coxarius, | 0 | 1 |
| Congestion of brain, | 1 | 2 | Old age, | 3 | 0 |
| Consumption of the lungs, | 11 | 1 | Palsy, | 1 | 0 |
| Convulsions, | 0 | 10 | Rupture of Aorta, | 1 | 0 |
| Diarrhœa, | 0 | 2 | Sore Mouth, | 0 | 1 |
| Dropsy, | 2 | 0 | Small pox, | 3 | 11 |
| —— head, | 1 | 2 | Still-born, | 0 | 9 |
| Disease of the Brain, | 2 | 0 | Tabes Mesenterica, | 0 | 1 |
| —— Heart, | 1 | 0 | Ulceration of Stomach, | 1 | 0 |
| —— Uterus | 1 | 0 | | — | — |
| Dysentery, | 2 | 0 | Total, 115— | 53 | 62 |
| Debility | 1 | 2 | | | |
| Enlargement of Liver, | 1 | 0 | | | |
| Effects of Laudanum, | 1 | 0 | | | |
| Fever, Remittent, | 3 | 1 | | | |
| —— Brain, | 1 | 0 | | | |
| —— Typhus, | 2 | 0 | | | |
| —— Typhoid, | 0 | 1 | | | |
| Gangrene, | 1 | 0 | | | |
| Hæmaturia, | 1 | 0 | | | |
| Inflammation of Breast, | 0 | 1 | | | |
| —— Brain, | 0 | 1 | | | |
| —— Bronchi, | 1 | 2 | | | |
| —— Lungs, | 1 | 5 | | | |
| —— Stomach, | 1 | 0 | | | |
| —— Liver, | 1 | 0 | | | |
| | — | — | | | |
| *Carried forward,* | 42 | 34 | | | |

Of the above, there were under 1 year, 22

| | |
|---|---|
| From 1 to 2 | 13 |
| 2 to 5 | 13 |
| 5 to 10 | 3 |
| 10 to 15 | 2 |
| 15 to 20 | 2 |
| 20 to 30 | 11 |
| 30 to 40 | 8 |
| 40 to 50 | 7 |
| 50 to 60 | 8 |
| 60 to 70 | 7 |
| 70 to 80 | 7 |
| 80 to 90 | 3 |
| 90 to 100 | 1 |
| 100 to 110 | 1 |
| Total, | 115 |

Of the above there were 8 from the almshouse, 12 people of colour, and 1 from the country, which are included in the total amount.

